# Responsive Ag@NiCo_2_O_4_ Nanowires Anchored on N-Doped Carbon Cloth as Array Electrodes for Nonenzymatic Glucose Sensing

**DOI:** 10.3390/molecules27227745

**Published:** 2022-11-10

**Authors:** Li Wang, Xiaowei Lv, Lei Zhang, Yanli Fang, Hui Wang, Jianwei Ren

**Affiliations:** 1 School of Chemistry & Environmental Engineering, Pingdingshan University, Pingdingshan 467000, China; 2 State Key Laboratory Base for Eco-Chemical Engineering, College of Chemical Engineering, Qingdao University of Science and Technology, Qingdao 266042, China; 3 Department of Mechanical Engineering Science, University of Johannesburg, Cnr Kingsway and University Roads, Auckland Park, Johannesburg 2092, South Africa

**Keywords:** Ag nanoparticles, NiCo_2_O_4_ nanowires, binder-free array electrode, glucose sensing

## Abstract

The development of responsive materials in a predictable manner is high on the list of the material industry’s trends. In this work, responsive Ag@NiCo_2_O_4_ nanowires were, firstly, anchored on N-doped carbon cloth (NC) and, then, employed as array electrodes for a nonenzymatic glucose-sensing application. The results showed that the highly conductive NiCo_2_O_4_ nanowires supported Ag nanoparticles and exhibited high conductivity and electrocatalytic properties. The fully exposed crystalline planes of Ag nanoparticles provided more active surface sites. As a result, the assembled Ag@NiCo_2_O_4_-NC electrodes for the glucose-sensing evaluation delivered a selectivity of 2803 μA mM^−1^ cm^−2^ and a detection limit of 1.065 μM, which outperformed the literature-reported Ag- and NiCo_2_O_4_-based glucose-sensing catalysts.

## 1. Introduction

The rapid and accurate monitoring of the blood glucose condition is an essential demand of diabetes care [1,2]. The enzymatic activity and measurement accuracy of commonly used enzymatic glucose sensors are subject to the performance of glucose oxidase, which, however, is sensitive to other variables, such as temperature, pH and other toxins. In recent decades, nonenzymatic glucose sensors based on the direct electrochemical oxidation of glucose have become a research focus because of their lower cost, higher selectivity and longer lifetime compared to enzyme glucose sensors [3,4]. In the literature, the performance of a nonenzymatic glucose sensor is determined using the specificity of the electrode on which the glucose oxidation occurs under catalysis [5]. In other words, the performance appraisal of electrochemical catalysts for glucose oxidation is key to the overall performance [6,7]. In this regard, although noble-metal (Pt, Ag, Au, Pd, etc.)-based catalysts were proved to be efficient and stable [8,9], their high costs have posed challenges for their wider use in glucose-sensing applications. To mitigate this issue, an economic solution was to load less noble metals onto catalysts, coupled with design features such as the surface morphology, particle size, distribution and choice of substrates [10,11,12]. In addition, binder-free electrodes with noble metals loaded onto porous substrates would exhibit a faster electron transfer compared to binder-based electrodes with higher resistances [13].

In this work, responsive Ag@NiCo_2_O_4_ nanowires were directly anchored on N-doped carbon cloth (NC) without the usage of any binders. The composite was then employed as an array electrode for a nonenzymatic glucose-sensing evaluation. As a result of the good conductivity of the NiCo_2_O_4_ nanowire substrates and well-exposed crystalline planes of Ag nanoparticles, the prepared Ag/NiCo_2_O_4_-NC composite exhibited good conductivity and excellent electrochemical activity for glucose-sensing applications.

## 2. Results and Discussions

### 2.1. Physical Characterization

**Figure 1:**
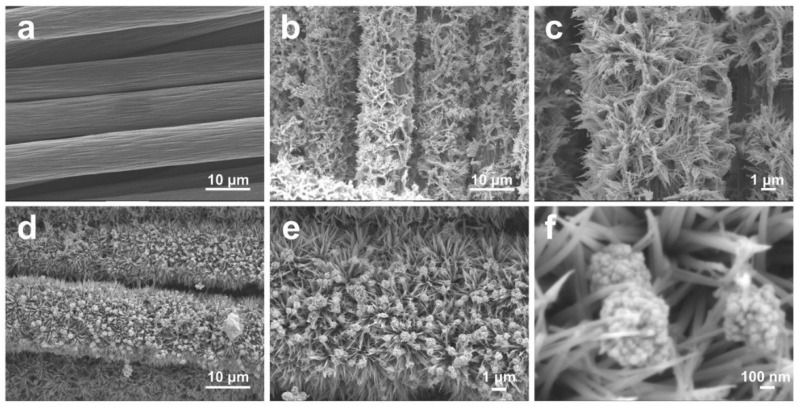
SEM images of samples of (**a**) CC, (**b**,**c**) NiCo_2_O_4_-NC and (**d**–**f**) Ag/NiCo_2_O_4_-NC.

Figure 1 shows the SEM images of the as-prepared samples. The smooth surface of the carbon cloth can be seen in Figure 1a. After being treated in a Tris-HCl solution (pH = 8.5) for 12 h, the DA molecules were, firstly, polymerized into a conformal PDA film on the carbon cloth substrate in a weak alkaline solution, and the sequential heat-treatment at 800 °C for 2 h promoted the rich nitrogen atoms in the PDA film to interact with the carbon networks in the carbon cloth. Eventually, the substrate of the nitrogen-doped carbon cloth (NC) was readily prepared [14]. Figure 1b,c exhibit the anchoring process of the uniform NiCo_2_O_4_ nanowires. After another PDA layer was coated onto the exterior of the NiCo_2_O_4_ nanowires, the inherent reducing ability of the PDA coupling with the UV irradiation yielded a Ag/NiCo_2_O_4_-NC sample (Figure 1d). It can be observed in Figure 1e,f that the conductive NiCo_2_O_4_ nanowires served as the ideal supports for the Ag nanoparticles. This, in turn, enabled the full exposure of different reactive planes and surface active sites of Ag nanoparticles to achieve the enhanced glucose-sensing capacities.

**Figure 2:**
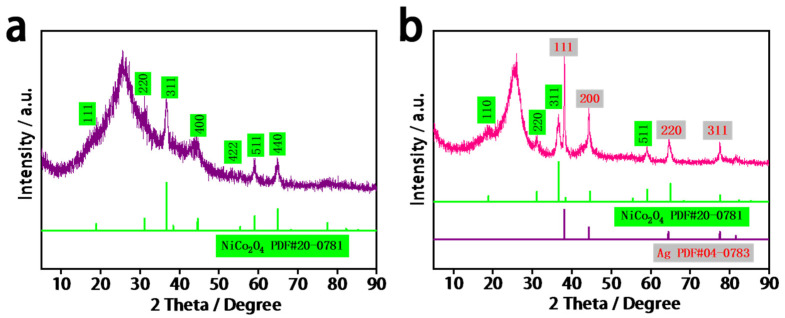
XRD patterns of samples of (**a**) NiCo_2_O_4_-NC and (**b**) Ag/NiCo_2_O_4_-NC.

Figure 2 shows the XRD patterns of the NiCo_2_O_4_-NC and Ag/NiCo_2_O_4_-NC samples. The diffraction peaks of the NiCo_2_O_4_-NC sample (Figure 2a) positioned at 18.9°, 31.4°, 36.7°, 44.6°, 59.1° and 64.98° could be individually assigned to the (111), (220), (311), (400), (511) and (440) planes of the NiCo_2_O_4_ nanowires, respectively (PDF#20-0781). After the loading of Ag nanoparticles (Figure 2b), in addition to the diffraction peaks of NiCo_2_O_4_ nanowires, four new peaks at 38.0°, 44.3°, 64.4° and 77.5° were found to belong to the (111), (200), (220) and (311) planes of Ag nanoparticles, respectively (PDF#04-0783). This observation confirmed the successful formation of a Ag/NiCo_2_O_4_-NC composite.

**Figure 3:**
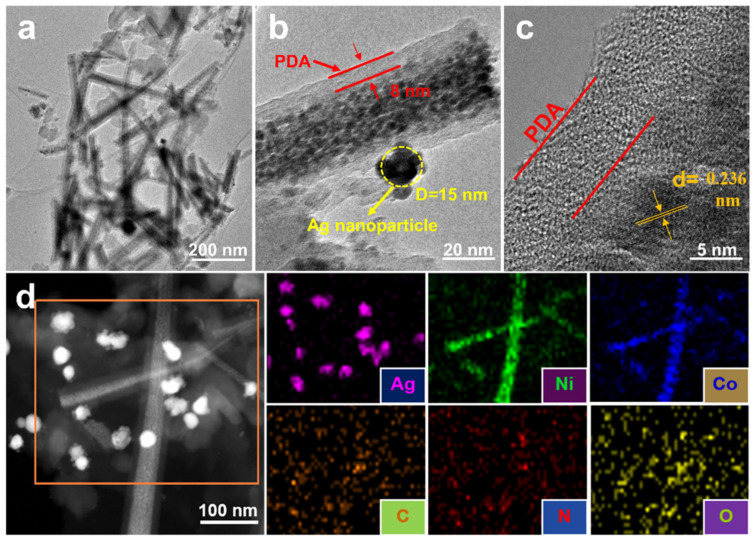
(**a**,**b**) TEM images, (**c**) HR-TEM image, (**d**) STEM image and elemental mapping of the corresponding region selected in figure d of Ag/NiCo_2_O_4_-NC sample.

Figure 3 shows the TEM, HR-TEM images and the elemental mapping of the Ag/NiCo_2_O_4_-NC sample. As can be clearly observed in Figure 3a–c, the NiCo_2_O_4_ nanowires were covered with PDA films with a thickness of 8 nm, and Ag nanoparticles in an approximate 15 nm size range were anchored on the NiCo_2_O_4_ nanowires. The fully exposed planes of Ag nanoparticles provided more reactive surface sites for glucose-sensing applications [15]. In the HR-TEM image in Figure 3c, the PDA film together with the lattice fringe of the Ag (111) crystalline plane were visible, with an interplant distance of 0.236 nm. The STEM image and EDX elemental mapping spectroscopies in Figure 3d confirmed the presence of Ag, Ni, Co, C, N and O elements. The percentage of elements in the detected region was 15.47 wt.% for Ag, 4.55 wt.% for Co, 9.10 wt.% for Ni, 41.22 wt.% for C, 12.45 wt.% for N and 17.21 wt.% for O.

**Figure 4:**
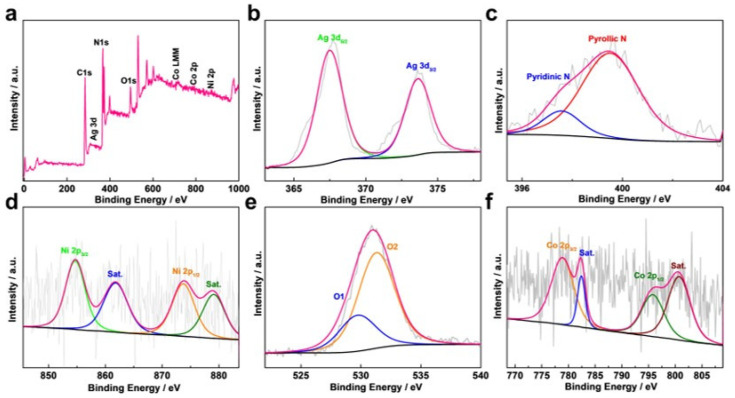
The XPS spectra of (**a**) Ag/NiCo_2_O_4_-NC sample; (**b**) Ag 3d, (**c**) N 1s, (**d**) Ni 2p, (**e**) O 1s and (**f**) Co 2p.

Figure 4 displays the XPS spectra of the Ag/NiCo_2_O_4_-NC sample. The signals of Ag 3d, N 1s, Ni 2p, O 1s and Co 2p are clearly visible in Figure 4a. Two peaks of Ag 3d_5/2_ and Ag 3d_3/2_ in Figure 4b appeared at the binding energies of 367.5 and 373.7 eV, respectively [16]. The rich N content of the PDA film in the Ag/NiCo_2_O_4_-NC sample was reflected both by the N 1s XPS signals of the pyrrolic N peak at 397.6 eV and the pyridinic N peak at 399.5 eV (Figure 4c) [17]. As reported in the literature, pyridinic N favorably improves the surface hydrophilicity and conductivity of catalysts by lowering the resistance of electron transfers [18,19]. Similarly, the Ni 2p XPS spectra in Figure 4d displayed two peaks at 873.7 eV and 854.7 eV, which were, sequentially, ascribed to Ni 2p_1/2_ and Ni 2p_3/2_ electron orbits, respectively. Two characteristic satellite peaks resulted from the surface oxidation [20]. From the O 1s XPS spectra in Figure 4e, two peaks of O1 (529.8 eV) and O2 (531.3 eV), individually, were shown to belong to the metal–O and physicochemical water bonds, respectively [21]. Two peaks of Co 2p_3/2_ and Co 2p_1/2_ were found (Figure 4f) at the binding energies of 778.8 eV and 795.6 eV, respectively. On top of that, two satellite peaks present at the binding energies of 782.4 eV and 800.7 eV resulted from the partial oxidation of Co^2+^ ions [22].

### 2.2. Electrochemical Measurements

**Figure 5:**
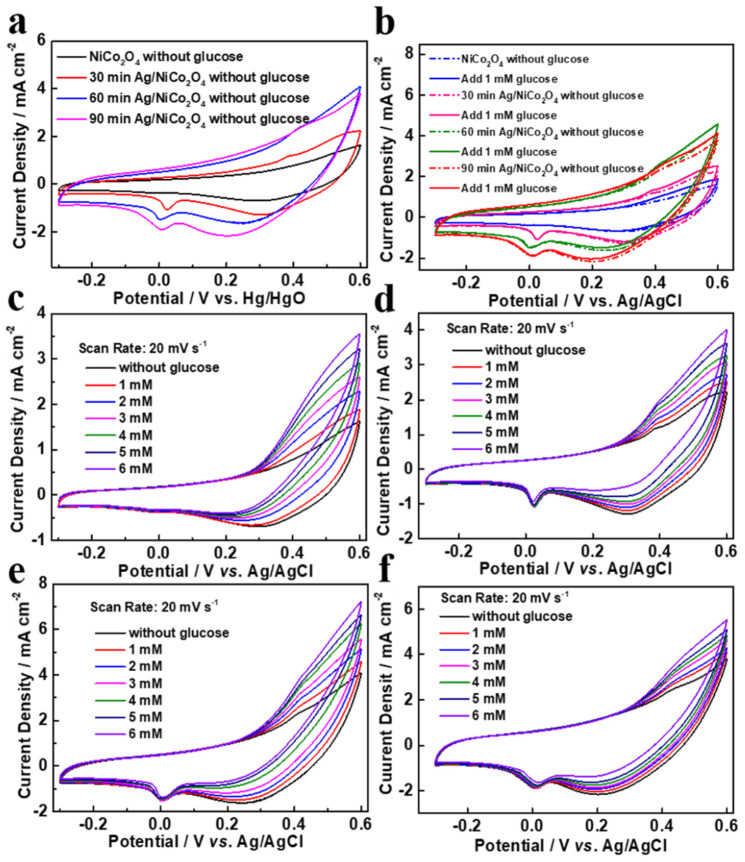
CV curves of different samples in (**a**) 0.1 M NaOH, (**b**) 0.1 M NaOH + 0.1 mM glucose at a scan rate of 20 mV s^−1^, (**c**) NiCo_2_O_4_-NC, (**d**) Ag/NiCo_2_O_4_-NC-30, (**e**) Ag/NiCo_2_O_4_-NC-60 and (**f**) Ag/NiCo_2_O_4_-NC-90 in 0.1 M NaOH and 1–6 mM glucose.

Figure 5 illustrates the CV curves of different samples in 0.1 M NaOH solution as electrolytes. As seen in Figure 5a, there was no reduction or oxidation peaks visible in the CV curve of the NiCo_2_O_4_-NC sample, which suggested that the NiCo_2_O_4_-NC sample did not deliver electrocatalytic activity in the 0.1 M NaOH solution. After the loading of Ag nanoparticles, the appearance of oxidation peaks at −0.3~0.60 V resulted from the oxidation of Ag^+^ on Ag/NiCo_2_O_4_-NC samples in 0.1 M NaOH. In contrast, in Figure 4b, the current densities increased after the addition of glucose, which indicated the electrocatalytic activity of the Ag/NiCo_2_O_4_-NC sample for glucose sensing [23]. Moreover, the larger current generated by the Ag/NiCo_2_O_4_-NC-60 sample compared with those of the Ag/NiCo_2_O_4_-NC-30 and Ag/NiCo_2_O_4_-NC-90 samples suggested that increasing the number of Ag nanoparticles favored glucose oxidation. It was noticed that the voltammetric responses of the NiCo_2_O_4_-NC and Ag/NiCo_2_O_4_-NC samples in Figure 5c–f increased linearly with the increasing concentrations of glucose in the concentration range of 1–6 mM, which could be attributed to the conversion between glucose and gluconic acid, as described in Equation (1).

CH_2_OH(CHOH)_4_CHO + Ag^+^ + OH^−^ → CH_2_OH(CHOH)_4_COO^−^ + Ag +H_2_O (1)

where the surface Ag^+^ ions were reduced to Ag nanoparticles using glucose, and, in turn, more glucose molecules were oxidized by driving the reaction to the right. Thus, more electrons were generated with the addition of glucose, which further led to the increase in the peak current. Overall, the electrocatalytic response from Ag/NiCo_2_O_4_-NC-90 sample was larger than that of the Ag/NiCo_2_O_4_-NC-30 and Ag/NiCo_2_O_4_-NC-60 samples.

**Figure 6:**
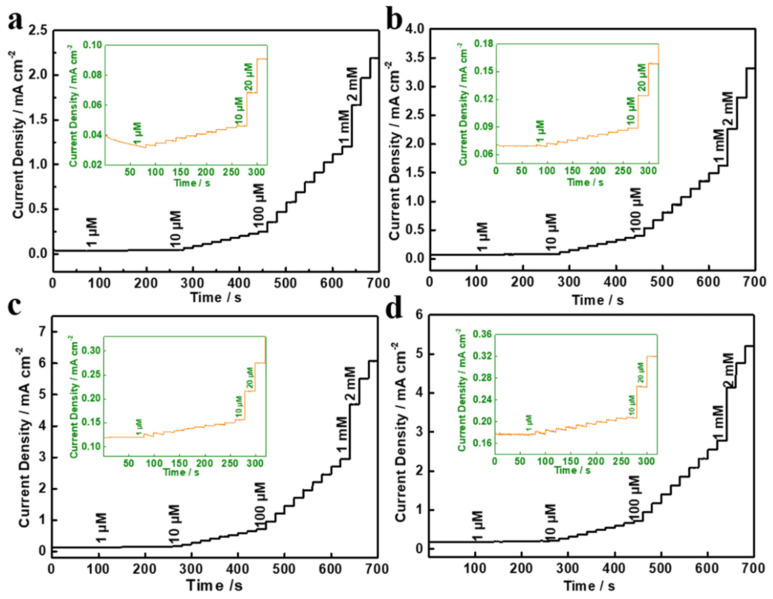
Typical amperometric response of different samples with the successive addition of glucose from 1 μM to 2 mM in 0.1 M NaOH solution: (**a**) NiCo_2_O_4_-NC, (**b**) Ag/NiCo_2_O_4_-NC-30, (**c**) Ag/NiCo_2_O_4_-NC-60 and (**d**) Ag/NiCo_2_O_4_-NC-90. Inset: the amperometric response to glucose concentration of 1–20 μM.

Figure 6 shows the amperometric responses of different samples on the successive addition of various concentrations of glucose from 1 μM to 2 mM in a 0.1 M NaOH solution at a working potential of 0.50 V (vs. Ag/AgCl). By comparison, it was clear that the Ag/NiCo_2_O_4_-NC samples delivered a quick response by reaching a steady current within 10 s, which suggested a high electrocatalytic activity and fast electron transfer for glucose sensing.

**Figure 7:**
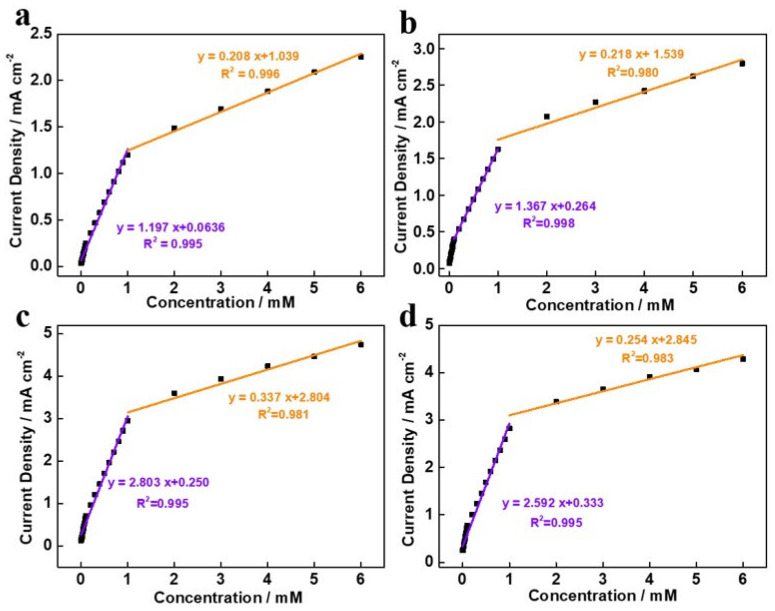
The corresponding calibration curves of different samples: (**a**) NiCo_2_O_4_-NC, (**b**) Ag/NiCo_2_O_4_-NC-30, (**c**) Ag/NiCo_2_O_4_-NC-60 and (**d**) Ag/NiCo_2_O_4_-NC-90.

Figure 7 depicts the calibration curves of the different as-prepared samples. As can be seen, the glucose assay responses were linear at 0.1–6 mM glucose concentrations with acceptable correlation coefficients for NiCo_2_O_4_-NC (R^2^ = 0.995), Ag-NiCo_2_O_4_-NC-30 (R^2^ = 0.998), Ag-NiCo_2_O_4_-NC-60 (R^2^ = 0.995) and Ag-NiCo_2_O_4_-NC-90 (R^2^ = 0.995). Apparently, this is also applicable for normal human blood glucose levels of 3–8 mM. Moreover, the sensitivities of samples of NiCo_2_O_4_-NC and Ag-NiCo_2_O_4_-NC-60 were 1197 μA mM^−1^ cm^−2^ and 2803 μA mM^−1^ cm^−2^, respectively, while their individual detection limits were 2.494 μM and 1.065 μM, respectively. Table 1 compares key parameters with other Ag- and NiCo_2_O_4_-based glucose sensors reported in the literature. The Ag-NiCo_2_O_4_-NC-60 sample exhibited the most promising results for nonenzymatic glucose-sensing applications through its outperformed appraisal. The good activity of the Ag/NiCo_2_O_4_-NC-60 sample for glucose oxidation could be ascribed to the following factors: firstly, the whole electrode had a binder-free structure that favored the electron transfer; secondly, the array-like structure with a large surface area benefitted the mass transfer and provided more active sites for the reaction; lastly, the uniform distribution of Ag nanoparticles exposed more catalytic sites for glucose oxidation.

**Figure 8:**
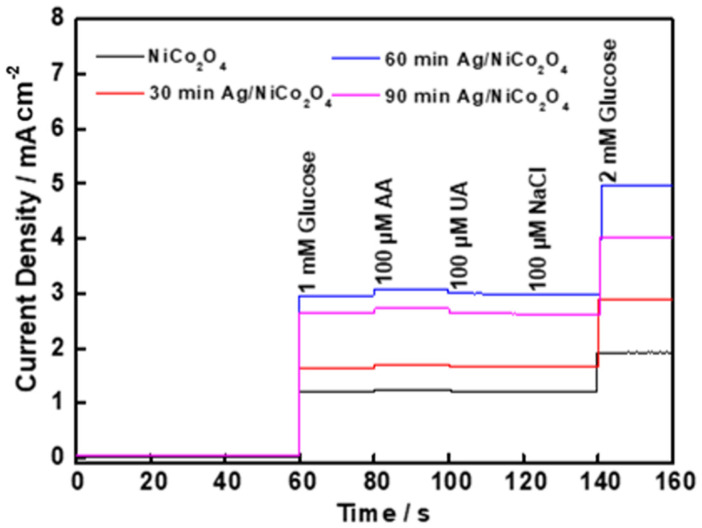
Amperometric i–t curve responses of NiCo_2_O_4_-NC and Ag/NiCo_2_O_4_-NC electrodes in 0.1 M NaOH with the presence of glucose (1 mM) and interfering compounds AA (100 μM), UA (100 μM) and NaCl (100 μM).

As it is known, interfering species in human blood, such as uric acid (UA), ascorbic acid (AA) and NaCl, can interfere with the satisfaction of glucose sensing. Therefore, the selectivity is another key parameter determining whether the developed catalysts can be widely incorporated into nonenzymatic glucose sensors. Based on the understanding that the concentration of glucose was at least 10 times higher than that of interfering species, glucose (1.0 mM), AA (100 μM), UA (100 μM) and NaCl (100 μM) were added sequentially into the 0.1 M NaOH solution at an applied potential of +0.50 V. As shown in Figure 8, the two assembled electrodes responded unexceptionally to the glucose oxidation, but only limited responses were detected after the addition of interfering species. This suggested a high selectivity and anti-interference properties of the NiCo_2_O_4_-NC and Ag/NiCo_2_O_4_-NC electrodes towards glucose sensing in alkaline media.

## 3. Materials and Methods

### 3.1. Preparation of N-Doped Carbon Cloth (NC)

All the chemicals used in this experiment were analytically pure and used directly without further purification. For the carbon cloth, a hydrophilic commercial carbon cloth (W0S1009) produced by carbon energy groups in Taiwan was selected, with a thickness of 0.33 mm and a unit weight of 120 g/m^2^. Firstly, the pH value of 10 mM tris(hydroxymethyl)aminomethane solution was adjusted to 8.5 by adding concentrated HCl. Then, a piece of carbon cloth (3 cm × 6 cm, Carbon Energy Technology Co., Ltd., Taiwan, China; type: WOS1011) was immersed into 160 mL of Tris solution in a flask to which ultrasound was applied to remove any bubbles. After that, 160 mg of dopamine (DA) was added into the flask under stirring for 12 h to polymerize into a conformal polydopamine (PDA) film on the carbon cloth substrate. Subsequently, the PDA-coated carbon cloth was taken out, rinsed with deionized water and dried. Lastly, the dried PDA-coated carbon cloth was placed in a furnace under a N_2_ atmosphere, which was heated up to 800 °C at 5 °C min^−1^ and maintained for 2 h to yield a N-doped carbon cloth (NC) sample.

### 3.2. Preparation of NiCo_2_O_4_-NC

Firstly, 1 mmol of Co(NO_3_)_2_·6H_2_O, 0.5 mmol of Ni(NO_3_)_2_·9H_2_O, 5 mmol of CH_4_N_2_O and 5 mmol of NH_4_F were dissolved together into 20 mL of deionized water. Then, the mixture was transferred to a 25 mL autoclave enclosed with a piece of carbon cloth (3 cm × 2 cm). After that, the autoclave was kept at 120 °C for 12 h to obtain the NiCo_2_O_4_-NC sample.

### 3.3. Preparation of Ag-NiCo_2_O_4_-NC

The NiCo_2_O_4_-NC sample prepared in Step 3.2 was, firstly, immersed in 25 mL of Tris solution and then 50 mg of DA was added under stirring. After 12 h, the resultant sample was collected, rinsed with deionized water, dried and cut into several equal smaller pieces (1 cm × 1 cm). Those pieces were separately put into different cuvettes containing 4 mL of 0.1 mmol/L AgCl solution and exposed to UV irradiation (254 nm) with different exposure times (30, 60, 90 min). The obtained Ag/NiCo_2_O_4_-NC samples were rinsed, dried and tested for electrochemical performance.

### 3.4. Electrochemical Measurements

The measurement of the electrochemical performance of the as-prepared samples was conducted on a CHI 660E electrochemical workstation within a three-electrode electrochemical cell containing Ag/AgCl as the reference electrode (saturated KCl solution), a platinum wire as the counter electrode and Ag/NiCo_2_O_4_-NC as the working electrode. A cyclic voltammetry (CV) measurement was carried out in a 0.1 M NaOH electrolyte, and the potential scan rate was 50 mV s^−1^ in the potential range of −0.3~0.60 V vs. Ag/AgCl. The electrode was, firstly, scanned in NaOH without the presence of glucose and was, subsequently, scanned in NaOH with glucose (Macklin reagent, A.R., Shanghai, China) added at concentrations of 50 μM, 100 μM, 500 μM, 1 mM, 2 mM, 3 mM, 4 mM and 5 mM.

### 3.5. Physical Characterization

The crystalline structure of the sample was analyzed using an X-ray diffraction (XRD, Shimadzu XD-3A (Japan) diffractometer, using a Cu Ka radiation operated at 40 kV and 35 mA). The morphology of the catalyst was observed with a Carl Zeiss Ultra Plus field emission scanning electron microscope (SEM) and transmission electron microscopy (TEM) measurements, which were carried out using a JEM-2010 Electron Microscope (Japan) with an acceleration voltage of 200 kV coupled with an energy-dispersive X-ray (EDX) analysis technique. X-ray photoelectron spectroscopy (XPS) was performed on a VG Escalab210 spectrometer with a Mg 300 W X-ray source.

## 4. Conclusions

In this work, responsive Ag@NiCo_2_O_4_ nanowires were anchored on N-doped carbon cloth (NC) via a PDA-film-mediated approach. The composite catalysts were employed as the array electrodes for the nonenzymatic glucose-sensing appraisal. The results showed that the prepared Ag/NiCo_2_O_4_-NC composite exhibited good electrocatalytic properties. This was attributed to the high conductivity of the NiCo_2_O_4_ nanowires used as substrates for the Ag nanoparticles and the fully exposed crystalline planes of Ag nanoparticles provided more surface reactive sites for glucose sensing. As a result, the assembled Ag/NiCo_2_O_4_-NC sensor delivered a selectivity of 2803 μA mM^−1^ cm^−2^ and a detection limit of 1.065 μM. The high selectivity further predicted the practical value of the Ag/NiCo_2_O_4_-NC catalyst in real applications.

## Figures and Tables

**Table 1:** Comparison of the key parameters of literature-reported Ag- and NiCo_2_O_4_-based glucose-sensing catalysts.

Catalyst	Sensitivity (μA cm^−2^ mM^−1^)	Linear Range (mM)	DOL (μM)	R^2^	Ref.
NiCo_2_O_4_	1197	0.001~1	2.494	0.995	This work
	208	1~6	14.356	0.996	
30 min Ag/NiCo_2_O_4_	1367	0.001~1	2.190	0.998	This work
	218	1~6	13.486	0.980	
60 min Ag/NiCo_2_O_4_	2803	0.001~1	1.065	0.995	This work
	337	1~6	8.733	0.981	
90 min Ag/NiCo_2_O_4_	2592	0.001~1	1.152	0.995	This work
	254	1~6	11.610	0.983	
NiO@Ag/GCE	67.51	0~1.28	1.01	0.994	[24]
NiCo_2_O_4_/ECF	1947.2	0.005~19.175	1.5	0.995	[25]
NiCo_2_O_4_@CNT	1424.41	0.01~1.55	1.14	0.993	[26]
Ag@ZIF-67/GCE	379	0.002~1	0.66	0.995	[16]
Ag-CuO/rGO/GCE	214.37	0.01~28	0.28	0.995	[27]
Ag/NiO/rGO	1869.4	--	--	0.996	[28]

## Data Availability

Data can be obtained upon written request to the corresponding author and with a proper justification.

## Sample Availability

Samples of the compounds are available from the authors.

## References

[1] Slaughter G., Kulkarni T. (2017). Highly Selective and Sensitive Self-Powered Glucose Sensor Based on Capacitor Circuit. Sci. Rep..

[2] Zhang E., Cao Z. (2018). Coated glucose sensors dodge recalibration. Nat. Biomed Eng..

[3] Jian M., Wang C., Wang Q., Wang H., Xia K., Yin Z., Zhang M., Liang X., Zhang Y. (2017). Advanced carbon materials for flexible and wearable sensors. Sci. China Mater..

[4] Li H., Liu C., Wang D., Zhang C. (2017). Chemiluminescence cloth-based glucose test sensors (CCGTSs): A new class of chemiluminescence glucose sensors. Biosens. Bioelectron..

[5] Shin D.H., Kim W., Jun J., Lee J.S., Kim J.H., Jang J. (2018). Highly selective FET-type glucose sensor based on shape-controlled palladium nanoflower-decorated graphene. Sens. Actuators B Chem..

[6] Cao M., Wang H., Ji S., Zhao Q., Pollet B.G., Wang R. (2019). Hollow core-shell structured Cu_2_O@Cu_1.8_S spheres as novel electrode for enzyme free glucose sensing. Mater. Sci. Eng. C.

[7] Cao M., Wang H., Kannan P., Ji S., Wang X., Zhao Q., Linkov V., Wang R. (2019). Highly efficient non-enzymatic glucose sensor based on Cu_x_S hollow nanospheres. Appl. Surf. Sci..

[8] Kim S.K., Jeon C., Lee G.H., Koo J., Cho S.H., Han S., Shin M.H., Sim J.Y., Hahn S.K. (2019). Hyaluronate-Gold Nanoparticle/Glucose Oxidase Complex for Highly Sensitive Wireless Noninvasive Glucose Sensors. ACS Appl. Mater. Interfaces.

[9] Qiu J., Wang X., Ma Y., Yu Z., Li T. (2021). Stretchable Transparent Conductive Films Based on Ag Nanowires for Flexible Circuits and Tension Sensors. ACS Appl. Nano Mater..

[10] Bihar E., Wustoni S., Pappa A.M., Salama K.N., Baran D., Inal S. (2018). A fully inkjet-printed disposable glucose sensor on paper. NPG Flexible Electronics.

[11] Huang H.J., Ning X., Zhou M.B., Sun T., Wu X., Zhang X.P. (2021). A Three-Dimensional Printable Liquid Metal-Like Ag Nanoparticle Ink for Making a Super-Stretchable and Highly Cyclic Durable Strain Sensor. ACS Appl. Mater. Interfaces.

[12] Liao Q.L., Jiang H., Zhang X.W., Qiu Q.F., Tang Y., Yang X.K., Liu Y.L., Huang W.H. (2019). A single nanowire sensor for intracellular glucose detection. Nanoscale.

[13] Wei M., Qiao Y., Zhao H., Liang J., Li T., Luo Y., Lu S., Shi X., Lu W., Sun X. (2020). Electrochemical non-enzymatic glucose sensors: Recent progress and perspectives. Chem. Commun..

[14] Lv X., Ji S., Lu J., Zhang L., Wang X., Wang H. (2021). Quick in situ generation of a quinone-enriched surface of N-doped carbon cloth electrodes for electric double-layer capacitors. Dalton Trans..

[15] Gong K., Hu Q., Xiao Y., Cheng X., Liu H., Wang N., Qiu B., Guo Z. (2018). Triple layered core–shell ZVI@carbon@polyaniline composite enhanced electron utilization in Cr(vi) reduction. J. Mater. Chem. A.

[16] Meng W., Wen Y., Dai L., He Z., Wang L. (2018). A novel electrochemical sensor for glucose detection based on Ag@ZIF-67 nanocomposite. Sensors Actuators B Chem..

[17] Lv X., Ji S., Linkov V., Wang X., Wang H., Rongfang W. (2020). Three-dimensional *N*-doped super-hydrophilic carbon electrodes with porosity tailored by Cu_2_O template-assisted electrochemical oxidation to improve performance of electrical double layer capacitors. J Mater. Chem. A.

[18] Yang X., Li Q., Lu E., Wang Z., Gong X., Yu Z., Guo Y., Wang L., Guo Y., Zhan W. (2019). Taming the stability of Pd active phases through a compartmentalizing strategy toward nanostructured catalyst supports. Nat. Commun..

[19] Zhang C., Fu L., Liu N., Liu M., Wang Y., Liu Z. (2011). Synthesis of nitrogen-doped graphene using embedded carbon and nitrogen sources. Adv. Mater..

[20] Zhang L., Wang H., Ji S., Wang X., Wang R. (2019). Porous-sheet-assembled Ni(OH)_2_/NiS arrays with vertical in-plane edge structure for supercapacitors with high stability. Dalton Trans..

[21] Chen F., Wang H., Ji S., Linkov V., Wang R. (2018). Core-shell structured Ni_3_S_2_@Co(OH)_2_ nano-wires grown on Ni foam as binder-free electrode for asymmetric supercapacitors. Chem. Eng. J..

[22] Boppella R., Tan J., Yang W., Moon J. (2018). Homologous CoP/NiCoP Heterostructure on N-Doped Carbon for Highly Efficient and pH-Universal Hydrogen Evolution Electrocatalysis. Adv. Func. Mater..

[23] Sun S., Zhang X., Sun Y., Zhang J., Yang S., Song X., Yang Z. (2013). A facile strategy for the synthesis of hierarchical CuO nanourchins and their application as non-enzymatic glucose sensors. RSC Adv..

[24] Song J., Xu L., Xing R., Qin W., Dai Q., Song H. (2013). Ag nanoparticles coated NiO nanowires hierarchical nanocomposites electrode for nonenzymatic glucose biosensing. Sens. Actuators B Chem..

[25] Liu L., Wang Z., Yang J., Liu G., Li J., Guo L., Chen S., Guo Q. (2018). NiCo_2_O_4_ nanoneedle-decorated electrospun carbon nanofiber nanohybrids for sensitive non-enzymatic glucose sensors. Sens. Actuators B Chem..

[26] Tang X., Zhang B., Xiao C., Zhou H., Wang X., He D. (2016). Carbon nanotube template synthesis of hierarchical NiCoO_2_ composite for non-enzyme glucose detection. Sens. Actuators B Chem..

[27] Xu D., Zhu C., Meng X., Chen Z., Li Y., Zhang D., Zhu S. (2018). Design and fabrication of Ag-CuO nanoparticles on reduced graphene oxide for nonenzymatic detection of glucose. Sensors Actuators B Chem..

[28] Ngo Y.-L.T., Hoa L.T., Chung J.S., Hur S.H. (2017). Multi-dimensional Ag/NiO/reduced graphene oxide nanostructures for a highly sensitive non-enzymatic glucose sensor. J. Alloys Compd..

